# Maternal, Fetal, and Neonatal Outcomes in Pregnant Dengue Patients in Mexico

**DOI:** 10.1155/2018/9643083

**Published:** 2018-01-21

**Authors:** Carlos Machain-Williams, Eric Raga, Carlos M. Baak-Baak, Sungmin Kiem, Bradley J. Blitvich, Celso Ramos

**Affiliations:** ^1^Laboratorio de Arbovirología, Centro de Investigaciones Regionales Dr. Hideyo Noguchi, Universidad Autónoma de Yucatán, Mérida, YUC, Mexico; ^2^Korean International Cooperation on Infectious Diseases (KOICID), Busan, Republic of Korea; ^3^Departamento de Atención Médica, Servicios de Salud de Veracruz-Coordinación Clínica Médica, Instituto Mexicano del Seguro Social, Xalapa, VER, Mexico; ^4^Department of Infectious Diseases, Inje University, Haeundae Paik Hospital, Busan, Republic of Korea; ^5^Department of Veterinary Microbiology and Preventive Medicine, College of Veterinary Medicine, Iowa State University, Ames, IA, USA; ^6^Centro de Investigación Sobre Enfermedades Infecciosas, Instituto Nacional de Salud Pública, Cuernavaca, MOR, Mexico

## Abstract

To increase our understanding of the consequences of dengue virus infection during pregnancy, a retrospective analysis was performed on the medical records of all completed pregnancies (live births and pregnancy losses) at nine public hospitals in the Gulf of Mexico from January to October 2013. Eighty-two patients developed clinical, laboratory-confirmed dengue virus infections while pregnant. Of these, 54 (65.9%) patients were diagnosed with dengue without warning signs, 15 (18.3%) patients were diagnosed with dengue with warning signs, and 13 (15.9%) patients had severe dengue. Five (38.5%) patients with severe dengue experienced fetal distress and underwent emergency cesarean sections. Four patients delivered apparently healthy infants of normal birthweight while the remaining patient delivered a premature infant of low birthweight. Patients died of multiple organ failure during or within 10 days of the procedure. Severe dengue was also associated with obstetric hemorrhage (30.8%, four cases), preeclampsia (15.4%, two cases), and eclampsia (7.7%, one case). These complications were less common or absent in patients in the other two disease categories. Additionally, nonsevere dengue was not associated with maternal mortality, fetal distress, or adverse neonatal outcomes. In summary, the study provides evidence that severe dengue during pregnancy is associated with a high rate of fetal distress, cesarean delivery, and maternal mortality.

## 1. Introduction

Dengue is the most prevalent mosquito-borne viral disease affecting humans. The causative agent is dengue virus (DENV; family Flaviviridae, genus* Flavivirus*), an* Aedes*-transmitted virus that occurs as four serotypes. Dengue is endemic in most, if not all, tropical and subtropical countries and about half of the world's population is considered to be at risk [1–3]. According to one recent estimate, DENV is responsible for 390 million infections worldwide each year, of which 96 million produce clinical manifestations [4]. In Mexico, an estimated 139,000 symptomatic dengue episodes occur each year [5].

The World Health Organization (WHO) previously classified dengue using three disease categories: dengue fever (DF), dengue hemorrhagic fever (DHF), and dengue shock syndrome [6]. Due to several shortcomings in the classification scheme, most notably the underestimation of disease severity in some patients [7, 8], the WHO revised their guidelines in 2009 [9]. Dengue cases are now classified as either dengue with or without warning signs or severe dengue. Dengue without warning signs presents as an acute febrile illness with at least two of the following: nausea/vomiting, rash, aches and pains, leukopenia, and a positive tourniquet test. Warning signs are defined as abdominal pain, persistent vomiting, fluid accumulation, mucosal bleeding, lethargy, liver enlargement, and increasing haematocrit with decreasing platelets, and at least one must be observed to fulfill the diagnosis of dengue with warning signs. Severe dengue is associated with severe plasma leakage, severe bleeding, or organ failure.

The health consequences of dengue during pregnancy are not well understood and studies that have investigated this issue have produced variable results [10, 11]. One reason for this is because small study cohorts (<50 pregnant dengue patients) have been used; exceptions include studies performed in Brazil, French Guiana, and Sudan [12–16]. In Mexico, one study has explored the consequences of dengue virus infection during pregnancy using a cohort of eight dengue patients [17]. Due to the limited information on the impact of dengue during pregnancy and prompted by the recent discovery that the closely related Zika virus is a cause of microcephaly [18, 19], a retrospective study was performed to assess the maternal, fetal, and neonatal outcomes in a cohort of pregnant dengue patients in the Gulf of Mexico.

## 2. Materials and Methods

### 2.1. Study Setting

Nine public hospitals in the Gulf of Mexico participated in the study. The hospitals are located in three states: Tabasco, Tamaulipas, and Veracruz (three hospitals per state). Participating hospitals are as follows: the Instituto Mexicano del Seguro Social (IMSS; Mexican Institute of Social Safety), Instituto de Seguridad Social al Servicio de los Trabajadores del Estado (ISSSTE; Institute of Social Safety to the Service of State Workers), and Secretaria de Salud (SSA; Secretariat of Health).

### 2.2. Study Population and Dengue Diagnosis

A retrospective analysis was performed on the medical records of all completed pregnancies (live births and pregnancy losses regardless of gestation time) from January 1 to October 31, 2013. Dates prior to the introduction of Zika virus into the Americas were selected because DENV and Zika virus produce overlapping clinical presentations and serological assays cannot readily distinguish between antibodies to these viruses. All patients who developed laboratory-confirmed dengue while pregnant were included in the study cohort. Patients were classified into three groups: dengue without warning signs, dengue with warning signs, and severe dengue [9]. A patient was considered to have a laboratory-confirmed dengue infection if DENV NS1 or IgM was detected in an acute or convalescent-phase sample. An infant was considered to be born during the maternal-fetal viremia period (MFVP) if the time between maternal dengue onset and labor was ≤15 days [16]. The policy at each participating hospital is that all pregnant women with symptoms consistent with dengue are tested for evidence of dengue virus infection.

### 2.3. Statistical Analyses

Continuous variables are presented as mean ± standard deviation (SD) and nominal variables are presented as percentages. Statistical analyses were performed using the IBM SPSS Statistics version 22 software for Windows (IBM Corporation, Armonk, NY). Results were considered significant when* P* ≤ 0.05. Data was tested for normality using the Kolmogorov-Smirnov test and homoscedasticity using Levene's test. Data with normal distributions were analyzed using one-way ANOVA. Data that did not meet the assumptions of normality and homogeneity of variances were analyzed using the nonparametric Kruskal-Wallis test. The mean arterial pressure was calculated using the formula [(2 × diastolic) + systolic]/3 [20] and analyzed by one-way ANOVA.

### 2.4. Bioethical Considerations

To protect patient identities, all data were blind-coded by medical personnel at the participating hospitals before being forwarded electronically to the investigators. None of the investigators had access to the names of any patients or any other identifiable private information. The study was approved by the bioethical committee of all of the participating medical institutions.

## 3. Results

The study population consisted of 82 patients who developed clinical, laboratory-confirmed DENV infections during pregnancy. Fifty-four (65.9%) patients were diagnosed with dengue without warning signs, 15 (18.3%) patients were diagnosed with dengue with warning signs, and 13 (15.9%) patients had severe dengue. Five (38.5%) patients with severe dengue died. The ages of patients in the entire sample population ranged from 16 to 36 years old (mean: 25.6 yo.). Age ranges of patients with dengue without warning signs, dengue with warning signs, and severe dengue were remarkably similar (Table 1). The patients lived in Veracruz (*n* = 32), Tabasco (*n* = 31), and Tamaulipas (*n* = 19). Forty-four patients were positive for DENV IgM and 37 patients were positive for DENV NS1. An additional patient was positive for both DENV IgM and NS1. Only six patients were tested using both assays.

Illness onset occurred in patients diagnosed with dengue without warning signs at 16.0 to 39.4 weeks of gestation (mean: 27.5 weeks; Table 1). Of these, 23 (42.6%) patients developed symptoms during the second trimester and 31 (57.4%) patients became symptomatic in the third trimester. Patients diagnosed with dengue with warning signs developed symptoms at 15.4 to 37.3 weeks of gestation (mean: 28.2 weeks). Seven (46.7%) patients developed symptoms during the second trimester and eight (53.3%) patients became symptomatic during the third trimester. All patients with severe dengue became symptomatic in the third trimester (range: 34.0 to 36.3 weeks; mean: 33.1 weeks). No patients developed symptoms in the first trimester of pregnancy or immediate postpartum period.

The most common symptoms among groups of patients with nonsevere dengue were fever, myalgia, arthralgia, headache, and nausea (Table 2). The aforementioned symptoms in addition to liquid escape (peripheral edema, pleural effusion, and/or ascites) were also common among patients with severe dengue. Obstetric hemorrhage occurred in five patients, including four (30.8%) with severe dengue. Four patients developed preeclampsia, including two (15.4%) patients with severe dengue. Eclampsia occurred in one (7.7%) patient with severe dengue.

Alanine aminotransferase (ALT) and aspartate transaminase (AST) levels were often elevated in patients from all three disease categories and were significantly higher in patients with severe dengue compared to both groups of patients with nonsevere dengue (Table 1). Raised ALT and AST levels were detected in 92.6% and 46.3% of patients with dengue without warning signs, respectively, 93.3% and 66.7% of patients with dengue with warning signs, and 92.3% and 92.3% of patients with severe dengue (data not shown). Direct bilirubin (DB), indirect bilirubin (IB), and lactate dehydrogenase (LDH) levels were often elevated in patients with severe dengue but were usually within the normal range for patients with nonsevere dengue (Table 1). Of the patients with severe dengue, 76.9%, 61.5%, and 69.2% had elevated levels of DB, IB, and LDH, respectively (data not shown). Decreased platelet counts were detected in patients from all disease categories (Table 1). The majority (96.3%) of patients with dengue without warning signs and all (100%) patients in the two other disease categories had reduced platelet counts (data not shown).

Maternal mortality occurred in five cases (Table 3), they all had been diagnosed with severe dengue. Acute fetal distress was detected in each patient in the third trimester of pregnancy and emergency cesarean surgeries were performed. Two patients died in surgery; the three other patients were admitted to intensive care immediately after the procedure but expired five to ten days later. All patients died as a result of multiple organ failure. Four pregnancies were full-term and infants were within the normal weight range (defined as 2500 to 4000 gm). One patient delivered a premature infant of low birth weight (LBW). Symptoms characteristic of dengue were not observed in any infants from the time of delivery until hospital discharge. Two infants were delivered during the MFVP and three infants were delivered outside of this period.

The eight surviving patients with severe dengue gave birth vaginally. All pregnancies were full-term, there were no signs of fetal distress, and all neonates were of normal birthweight and apparently healthy. One (12.5%) infant was delivered during the MFVP. All patients with nonsevere dengue also experienced full-term pregnancies, gave birth vaginally, and delivered apparently healthy infants of normal birthweight. Two (3.7%) neonates delivered by patients with dengue without warning signs were born during the MFVP. No neonates from patients diagnosed with dengue with warning signs were delivered during this period.

## 4. Discussion

Medical records of 82 pregnant dengue patients collected from hospitals located in the Gulf of Mexico were retrospectively examined to increase our understanding of the consequences of symptomatic DENV infection during pregnancy. The study, which is one of the largest of its kind, provides evidence that severe dengue is associated with a high rate of fetal distress and cesarean delivery. Other studies have also provided evidence that dengue is associated with a high rate of cesarean delivery [13, 21–23]. Two of 10 (20%) DF patients and four of 16 (25%) DHF patients in Sri Lanka delivered infants by cesarean section [23]. One of six (16.7%) DF patients and one of two (50%) DHF patients in India also underwent cesarean surgery [22]. Cesarean sections were often performed on DHF, and to a lesser extent DF, patients described in case reports [24–30]. A notable difference between our study and most of the aforementioned studies is that cesarean surgery was not performed on any patients with nonsevere dengue included in this study. This could be due to differences in the clinical or laboratory guidelines used for dengue diagnosis in each study, serotypes or genotypes responsible for the infections, immunological variations between populations, or differences in medical practices among hospitals.

One (1.2%) patient, a 29-year-old woman designated ARS-05, delivered a LBW infant. Other studies also report an association between LBW and maternal dengue but at rates considerably higher than reported here [13, 16, 23, 31–33]. In Colombia, four (18.2%) of 22 infants delivered by patients with probable or suspected dengue and none of the infants delivered by 24 nondengue patients were LBW [32]. Six of 53 (11.3%) pregnant dengue patients in French Guiana and 4 of 26 (15.4%) pregnant dengue patients in Sri Lanka also delivered LBW infants [13, 23]. Many of the aforementioned LBW cases were attributed to preterm delivery and not intrauterine growth restriction. In this regard, ARS-05 was the only patient in our study who delivered a premature infant. In Brazil, 22 of 336 (6.5%) infants delivered by dengue patients were LBW [16]. The incidence of LBW was dramatically higher among infants delivered during the MFVP compared to those delivered outside of this period (22.0% and 4.4%, resp.). In our study, five infants were delivered during the MFVP and all were of normal birthweight. Therefore, one explanation for the low incidence of LBW is that few infants were delivered during the MFVP.

Dengue with or without warning signs was not associated with any apparent adverse maternal outcomes aside from the presence of symptoms typical of nonsevere dengue in the general population. Two (2.9%) cases of preeclampsia and one (1.5%) case of obstetric hemorrhage occurred among the 69 patients with nonsevere dengue but these rates are not higher than that observed during normal pregnancy [34, 35]. Eclampsia was not reported in any patients with nonsevere dengue. Among the patients with severe dengue, there was one (7.7%) case of eclampsia, two (15.4%) cases of preeclampsia, and four (30.8%) cases of obstetric hemorrhage. These rates are considerably higher than those during normal pregnancy [34, 35]. Several case reports have highlighted the occurrence of preeclampsia and eclampsia in pregnant DF and DHF patients [25, 28, 30, 36, 37]. Hemorrhage during labor occurred in five of 53 (9.3%) pregnant DF patients in French Guiana [13].

No fetal malformations were identified in our study, consistent with earlier reports [13, 23, 38–40]. We also provide no evidence that dengue is associated with fetal or perinatal death. This finding is also consistent with other studies but there are a few exceptions [13, 23, 41]. For example, four (25%) cases of fetal/neonatal death occurred in a cohort of 16 pregnant dengue patients in India [41].

None of the patients in our study cohort developed dengue symptoms in the first trimester. Patients with nonsevere dengue became symptomatic in the second or third trimesters and all patients with severe dengue developed symptoms in the third semester. A similar trend has been reported in other studies. Three of 53 (5.7%) patients in French Guiana developed dengue in the first trimester of pregnancy; all others (94.3%) became symptomatic in the second or third trimesters [13]. In Sri Lanka, one of 26 (3.8%) patients developed dengue in the first trimester; all others (96.2%) became first symptomatic in the second or third trimesters or postpartum period [23]. The reason(s) why illness onset usually occurs in mid to late pregnancy are unclear but it could be because dengue is not diagnosed as easily in early pregnancy or because the physiological or immunological changes that occur in mid to late pregnancy result in an increase in dengue virus susceptibility. Another explanation is that patients may be less likely to report or seek medical care for a febrile illness in early pregnancy compared to those with more advanced pregnancies. Some patients may not have been aware of their pregnancies in the first several weeks after conception and, therefore, are less likely to recall a febrile episode compared to a patient with a known pregnancy.

Many studies have described maternal to fetal transmission of DENV [27, 36, 38, 42–45]. In a review by Pouliot and colleagues [11], vertical transmission rates were calculated using information from 19 case reports and nine case series [11]. Sixteen of 25 (64.0%) infants in case reports and 18 of 143 (12.6%) infants in case series acquired DENV in utero. Twenty-nine of the 34 (85.3%) DENV-positive infants were symptomatic. Dengue symptoms were not observed in any neonates in our study. This could be considered unexpected given that many other studies have provided evidence of vertical transmission. One explanation for the lack of apparent vertical transmission is that not all of the medical personnel at the participating hospitals are aware that DENV can be vertically transmitted and, thus, vigilant monitoring for dengue symptoms in neonates was not always performed. We consider this to be unlikely because the Gulf of Mexico is a dengue-endemic region and local medical care providers are experienced in the clinical diagnosis of dengue. A subset of infants (e.g., those delivered by patients who developed dengue in the last few days of their pregnancies) may have become symptomatic after hospital discharge. Access to medical records that documented the health status of infants after discharge could not be obtained. A few other studies have also provided no evidence of maternal to fetal transmission of DENV [25, 26, 32]. For example, none of the 22 neonates delivered by dengue patients in Colombia displayed symptoms characteristic of dengue [32]. It is also important to note that diagnostic testing for DENV was not performed on neonates or umbilical cord blood and, therefore, asymptomatic infections that may have occurred in our study would not have been detected.

ALT and AST levels were elevated in the majority of patients from all three disease categories and were usually higher in patients with severe dengue compared to nonsevere dengue. Similar findings were reported in Sri Lanka with raised ALT and AST levels detected in 60% of pregnant DF patients and 100% of pregnant DHF patients [23]. Abnormal hepatic function is also a common feature of dengue in nonpregnant patients [46–49]. Platelet counts were reduced in almost all of the patients in our study cohort. Others have also reported low platelet counts in pregnant dengue patients [13, 23, 31]. It has also been documented that platelet counts are often reduced in nonpregnant dengue patients [46, 50].

In summary, we describe the maternal, fetal, and neonatal consequences of dengue during pregnancy using one of the largest cohorts for a study of this nature. We provide evidence that severe dengue during pregnancy is associated with a high risk of fetal distress, caesarean delivery, and maternal mortality and potentially an increased risk of obstetric hemorrhage, preeclampsia, and eclampsia. In contrast, nonsevere dengue was not associated with any apparent adverse maternal, fetal, or neonatal outcomes aside of the presence of symptoms characteristic of dengue in the general population.

## Figures and Tables

**Table 1:** Demographic, clinical, and laboratory information for a cohort of pregnant dengue patients in Mexico.

Parameters	Disease classification			*P *value
	Dengue without warning signs (*n* = 54)	Dengue with warning signs (*n* = 15)	Severe dengue (*n* = 13)	
	Mean (± standard deviation)			
*General information*				
Age (years)	25.7 ± 5.4	26.5 ± 5.4	24.2 ± 5.4	^a^≥0.05
Illness onset (weeks of gestation)	27.5 ± 6.1	28.2 ± 6.9	33.1 ± 5.5	^a^0.015
Time in hospital (days)	5.9 ± 1.4	6.3 ± 2.3	9.1 ± 4.2	^b^0.009
^c^ *Physiological parameters*				
Arterial pressure (mm Hg)	55.4 ±16.8	59.2 ± 15.4	71.8 ± 23.2	^b^0.013
Breathing rate (breaths per min.)	18.2 ±1.7	18.0 ± 0.8	19.4 ± 2.0	^b^≥0.05
Heart rate (beats per min.)	91.2 ±4.4	90.2 ± 5.4	96.3 ± 15.8	^b^≥0.05
^c,d^ *Blood count*				
Coagulation time: PT (sec) [12.1–15.0]	12.8 ± 1.9	13.0 ± 0.6	15.2 ± 3.6	^b^0.042
Coagulation time: PPT (sec) [22.7–33.7]	32.5 ± 5.1	33.8 ± 3.4	52.6 ± 74.6	^b^≥0.05
Hematocrit (%) [38–47]	36.5 ± 2.0	35.3 ± 2.4	36.1 ± 11.8	^b^0.018
Hemoglobin (g/dl) [12–16]	11.9 ± 0.7	11.6 ± 0.7	11.0 ± 1.2	^a^0.001
Leukocytes (×1000) (per *µ*l) [4–11]	4.3 ± 0.6	4.7 ± 0.8	5.9 ± 1.6	^b^0.001
Platelets (×1000) (per *µ*l) [150–450]	114.0 ± 20.1	113.8 ± 15.4	82.1 ± 41.8	^b^≥0.05
^c,d^ *Metabolic profile*				
Creatinine (mg/dL) [0.5–1.5]	1.0 ± 0.3	0.8 ± 0.5	1.3 ± 0.8	^b^≥0.05
Glucose (mg/dL) [60–120]	91.7 ± 12.3	93.4 ± 6.2	103.2 ± 21.0	^b^≥0.05
Urea (mg/dL) [15.0–38.0]	16.6 ± 8.3	13.7 ± 6.5	27.0 ± 20.9	^b^≥0.05
^c,d^ *Hepatic profile*				
ALB (g/dl) [3.0–5.0]	2.9 ± 0.2	4.1 ± 5.5	4.4 ± 6.8	^b^0.000
ALT (IU/L) [10–40]	54.6 ± 44.1	62.5 ± 68.1	261.9 ± 329.3	^b^0.001
AST (IU/L) [9–48]	85.6 ± 63.4	107.9 ± 125.1	469.0 ± 702.8	^b^0.000
DB (mg/dl) [0.1–0.3]	0.2 ± 0.2	0.2 ± 0.2	1.2 ± 1.3	^b^0.000
IB (mg/dl) [0.1–0.4]	0.2 ± 0.2	0.3 ± 0.2	0.8 ± 0.7	^b^0.000
LDH (UI/L) [140–280]	158.9 ± 114.0	242.9 ± 190.4	731.1 ± 752.1	^b^0.000

^a^One-way ANOVA test. ^b^Nonparametric Kruskal-Wallis test, ^c^determined during the acute-phase of illness, and ^d^normal ranges are provided in square brackets. ALB: albumin, ALT: alanine aminotransferase, AST: aspartate aminotransferase, DB: direct bilirubin, IB: indirect bilirubin, LDH: lactate dehydrogenase, PT: Prothrombin time, and PPT: Prothrombin partial time.

**Table 2:** Symptoms reported in a cohort of pregnant dengue patients in Mexico.

Symptom	Disease classification		
	Dengue without warning signs (*n* = 54)	Dengue with warning signs (*n* = 15)	Severe dengue (*n* = 13)
	Number (%)		
Abdominal pain	15 (27.8)	1 (6.7)	7 (53.8)
Arthralgia	44 (81.5)	13 (86.7)	9 (69.2)
Cough/diarrhea/conjunctivitis	8 (14.8)	5 (33.3)	2 (15.4)
Eclampsia	0 (0)	0 (0)	1 (7.7)
Edema/pleural effusion/ascites	5 (9.2)	0 (0)	9 (69.2)
Exanthema	19 (35.2)	1 (6.7)	4 (30.8)
Fever	54 (100.0)	15 (100.0)	13 (100.0)
Gingivorrhagia	0 (0)	8 (53.3)	3 (23.1)
Headache	35 (64.8)	14 (93.3)	10 (76.9)
Hepatomegaly/visceral damage	0 (0)	0 (0)	3 (23.1)
Hyporexia	8 (14.8)	3 (20.0)	4 (30.8)
Multiple organ failure	0 (0)	0 (0)	5 (38.5)
Myalgia	45 (83.3)	13 (86.7)	8 (61.5)
Nausea	23 (42.6)	9 (60.0)	8 (61.5)
Obstetric hemorrhage	1 (1.9)	0 (0)	4 (30.8)
Oral intolerance	17 (31.5)	1 (6.7)	4 (30.8)
Petechiae	14 (25.9)	3 (20)	5 (38.5)
Pneumonia	0 (0)	0 (0)	1 (7.7)
Preeclampsia	1 (1.9)	1 (6.7)	2 (15.4)
Rash	4 (7.4)	0 (0)	0 (0)
Retroocular pain	4 (7.4)	5 (33.3)	6 (46.2)
Shock	0 (0)	0 (0)	3 (23.1)

**Table 3:** Demographic characteristics, clinical manifestations, and other medical information for the deceased patients.

Patient information	Patient ID				
	SNI-04	ARS-05	GRKM-06	NCRE-69	PRTM1-100
*General information*					
Age (years)	28	29	18	17	16
State of residence	Veracruz	Veracruz	Veracruz	Veracruz	Tabasco
Illness onset (weeks/days of gestation)	37/2	33/0	39/0	37/0	35/6
Hospitalized (weeks/days of gestation)	37/5	33/4	39/1	37/4	36/2
Cesarean (weeks/days of gestation)	38/2	34/2	39/4	38/0	37/1
Death (number of days after surgery)	7	5	10	0	0
Total hospitalization time (days)	11	10	13	3	6
Premature birth (<37 weeks)	^a^−	^b^+	−	−	−
Birthweight (gm)	2750	1970	3050	2870	2815
Delivery during the MFVP	+	−	+	−	−
*Clinical manifestations*					
Abdominal pain	+	+	−	−	+
Arthralgia	+	+	−	+	−
Cough/diarrhea/conjunctivitis	−	−	−	−	+
Eclampsia	−	−	−	−	−
Edema/pleural effusion/ascites	+	+	+	+	+
Exanthema	−	−	−	+	−
Fever	+	+	+	+	+
Gingivorrhagia	−	+	−	−	−
Headache	+	+	+	−	+
Hepatomegaly/visceral damage	+	+	+	−	+
Hyporexia	+	−	−	−	−
Multiple organ failure	+	+	+	+	+
Myalgia	+	+	−	+	−
Nausea	+	−	+	−	+
Obstetric hemorrhage	−	−	+	+	+
Oral intolerance	+	+	−	−	−
Petechiae	−	+	+	−	−
Pneumonia	+	−	−	−	−
Preeclampsia	−	+	−	−	−
Rash	−	−	−	−	−
Retroocular pain	+	+	+	+	−
Shock	−	−	+	+	+
Shock onset (expressed as the number of days after the onset of dengue)	^c^N/A	N/A	7	3	6

^a^No, ^b^yes, and ^c^not applicable.
